# Scavenger Receptors Mediate the Role of SUMO and Ftz-f1 in *Drosophila* Steroidogenesis

**DOI:** 10.1371/journal.pgen.1003473

**Published:** 2013-04-18

**Authors:** Ana Talamillo, Leire Herboso, Lucia Pirone, Coralia Pérez, Monika González, Jonatan Sánchez, Ugo Mayor, Fernando Lopitz-Otsoa, Manuel S. Rodriguez, James D. Sutherland, Rosa Barrio

**Affiliations:** 1 CIC bioGUNE, Derio, Bizkaia, Spain; 2 IKERBASQUE, Basque Foundation for Science, Bilbao, Spain; University of California San Francisco, United States of America

## Abstract

SUMOylation participates in ecdysteroid biosynthesis at the onset of metamorphosis in *Drosophila melanogaster*. Silencing the *Drosophila* SUMO homologue *smt3* in the prothoracic gland leads to reduced lipid content, low ecdysone titers, and a block in the larval–pupal transition. Here we show that the SR-BI family of Scavenger Receptors mediates SUMO functions. Reduced levels of Snmp1 compromise lipid uptake in the prothoracic gland. In addition, overexpression of Snmp1 is able to recover lipid droplet levels in the *smt3* knockdown prothoracic gland cells. Snmp1 expression depends on Ftz-f1 (an NR5A-type orphan nuclear receptor), the expression of which, in turn, depends on SUMO. Furthermore, we show by *in vitro* and *in vivo* experiments that Ftz-f1 is SUMOylated. RNAi–mediated knockdown of *ftz-f1* phenocopies that of *smt3* at the larval to pupal transition, thus Ftz-f1 is an interesting candidate to mediate some of the functions of SUMO at the onset of metamorphosis. Additionally, we demonstrate that the role of SUMOylation, Ftz-f1, and the Scavenger Receptors in lipid capture and mobilization is conserved in other steroidogenic tissues such as the follicle cells of the ovary. *smt3* knockdown, as well as *ftz-f1* or Scavenger knockdown, depleted the lipid content of the follicle cells, which could be rescued by Snmp1 overexpression. Therefore, our data provide new insights into the regulation of metamorphosis via lipid homeostasis, showing that *Drosophila* Smt3, Ftz-f1, and SR-BIs are part of a general mechanism for uptake of lipids such as cholesterol, required during development in steroidogenic tissues.

## Author Summary

Steroid hormones are cholesterol derivates that control many aspects of animal physiology, including development of the adult organisms, growth, energy storage, and reproduction. In insects, pulses of the steroid hormone ecdysone precede molting and metamorphosis, the regulation of hormonal synthesis being a crucial step that determines animal viability and size. Reduced levels of the small ubiquitin-like modifier SUMO in the prothoracic gland block the synthesis of ecdysone, as SUMO is needed for cholesterol intake. Here we show that SUMO is required for the expression of Scavenger Receptors (Class B, type I). These membrane receptors are necessary for lipid uptake by the gland. Strikingly, their expression is sufficient to recover lipid content when SUMO is removed. The expression of the Scavenger Receptors depends on Ftz-f1, a nuclear transcription factor homologous to mammalian Steroidogenic factor 1 (SF-1). Interestingly, the expression of Ftz-f1 also depends on SUMO and, in addition, Ftz-f1 is SUMOylated. This modification modulates its capacity to activate the Scavenger Receptor Snmp1. The role of SUMO, Scavenger Receptors, and Ftz-f1 on lipid intake is conserved in other tissues that synthesize steroid hormones, such as the ovaries. These factors are conserved in vertebrates, with mutations underlying human disease, so this mechanism to regulate lipid uptake could have implications for human health.

## Introduction

Larval molting and metamorphosis in *Drosophila melanogaster* relies on pulses of ecdysteroid hormones. During the larval stages, the prothoracic gland (PG) is the tissue responsible for the synthesis of the steroid hormone ecdysone that is secreted to the hemolymph and converted to 20-hydroxyecdysone (20E) in target tissues [1]. Other tissues releasing ecdysteroids in the adult are the gonads, ovaries and testes. Cholesterol is the precursor of all steroid hormones. In arthropods, which are unable to synthesize cholesterol, ecdysteroids are synthesized from dietary cholesterol or phytosteroids. Cholesterol is converted to 20E through a series of enzymatic reactions that involve the cytochrome P450 enzymes coded by the Halloween genes *spook*, *spookier*, *phantom* (*phm*), *disembodied* (*dib*), *shadow*, *shade* and the Rieske non-heme iron oxigenase gene *neverland* [2], [3]. A transcriptional cascade triggered by 20E occurs at the onset of metamorphosis that leads to the sequential expression pattern of the transcription factors DHR3, Ftz-f1, E74 and E75 [4]. A similar transcriptional cascade is required during embryogenesis and could also be required for each larval molting [5]. However, many of the aspects involved in the regulation of ecdysteroid biosynthesis remain unknown.

The conjugation of SUMO (Small Ubiquitin-related MOdifier) to target proteins is a reversible post-translational modification highly conserved in all eukaryotic organisms. SUMOylation regulates diverse cellular processes including cell survival and proliferation, nuclear import, intracellular trafficking, transcriptional regulation and maintenance of genomic and nuclear integrity [6]. In addition, Smt3, the only SUMO homologue in *Drosophila*, has a role in the regulation of ecdysteroid levels during post-embryonic development [7]. Smt3 is required in the PG to produce the ecdysteroid peak necessary for the larval to pupal transition. Interestingly, *smt3* knockdown PG cells results in reduced intracellular channels and, as a consequence, exhibit low levels of lipid and sterol droplets indicating that impaired cholesterol uptake could contribute to the low ecdysteroid levels observed.

The nuclear hormone receptor superfamily function as transcription factors that regulate several functions such as metabolism, development and homeostasis. Recent studies have implicated the *Drosophila* nuclear receptors DHR96 and dHNF4 in cholesterol and triacylglycerol homeostasis and in lipid mobilization and fatty acids β-oxidation [8]–[11]. However, it is unknown whether the nuclear receptors might regulate cholesterol homeostasis in the PG. The mammalian NR5A2 Liver receptor homolog 1 (LRH-1), member of the Ftz-f1 subfamily of nuclear receptors, has been shown to be involved in lipid absorption and homeostasis [12]. In addition, LRH-1 and its close relative Steroidogenic Factor 1 (SF-1 or NR5A1) are modified by SUMO and also bind phospholipids [13]–[20]. Recently, the disruption of SF-1 SUMOylation in mice showed the inappropriate activation of target genes that led to endocrine abnormalities and changes in cell fate [21]. Interestingly, SF-1 regulates the expression of proteins related to sterol uptake and/or mobilization such as the Scavenger Receptor Class B type I (SR-BI), which belongs to the Cluster of Differentiation 36 (CD36) family [22], [23]. SR-BI, in addition to its role in the selective uptake of High Density Lipoprotein cholesteryl ester, is required for the formation of the microvillar channels in the mammalian adrenal gland [24]. SF-1 and its *Drosophila* orthologue Ftz-f1 control the transcriptional regulation of cytochrome P450 enzymes involved in sterol conversion, and therefore could play similar roles in the activation of steroid synthesis [25], [26].

In order to clarify the role of SUMOylation in the mechanism of cholesterol uptake, we analyzed the function of the *Drosophila* CD36 family and Ftz-f1 in the PG during steroidogenesis at the onset of pupariation. We show that the *Drosophila* CD36 family member Sensory neuron membrane protein (Snmp1) is necessary for lipid uptake in the PG, downstream of Smt3 function. We also show that SUMO is required for *ftz-f1* expression and, in addition, Ftz-f1 is modified by SUMO *in vitro* and *in vivo*. More importantly, reduced levels of Ftz-f1 in the PG leads to impaired pupariation with reduced levels of lipid droplets, similar to the *smt3* knockdown phenotype. Conversely, overexpression of *ftz-f1* is able to rescue Snmp1 expression in *smt3* knockdown PGs. Finally, extending our observations in the PG, we saw that Smt3, Ftz-f1 and the Scavenger Receptors have a role in lipid uptake during ovarian steroidogenesis. Our results suggest similar requirements for cholesterol uptake in various steroidogenic tissues.

## Results

### *ftz-f1* knockdown in the PG phenocopies *smt3i* phenotype

**Figure 1:**
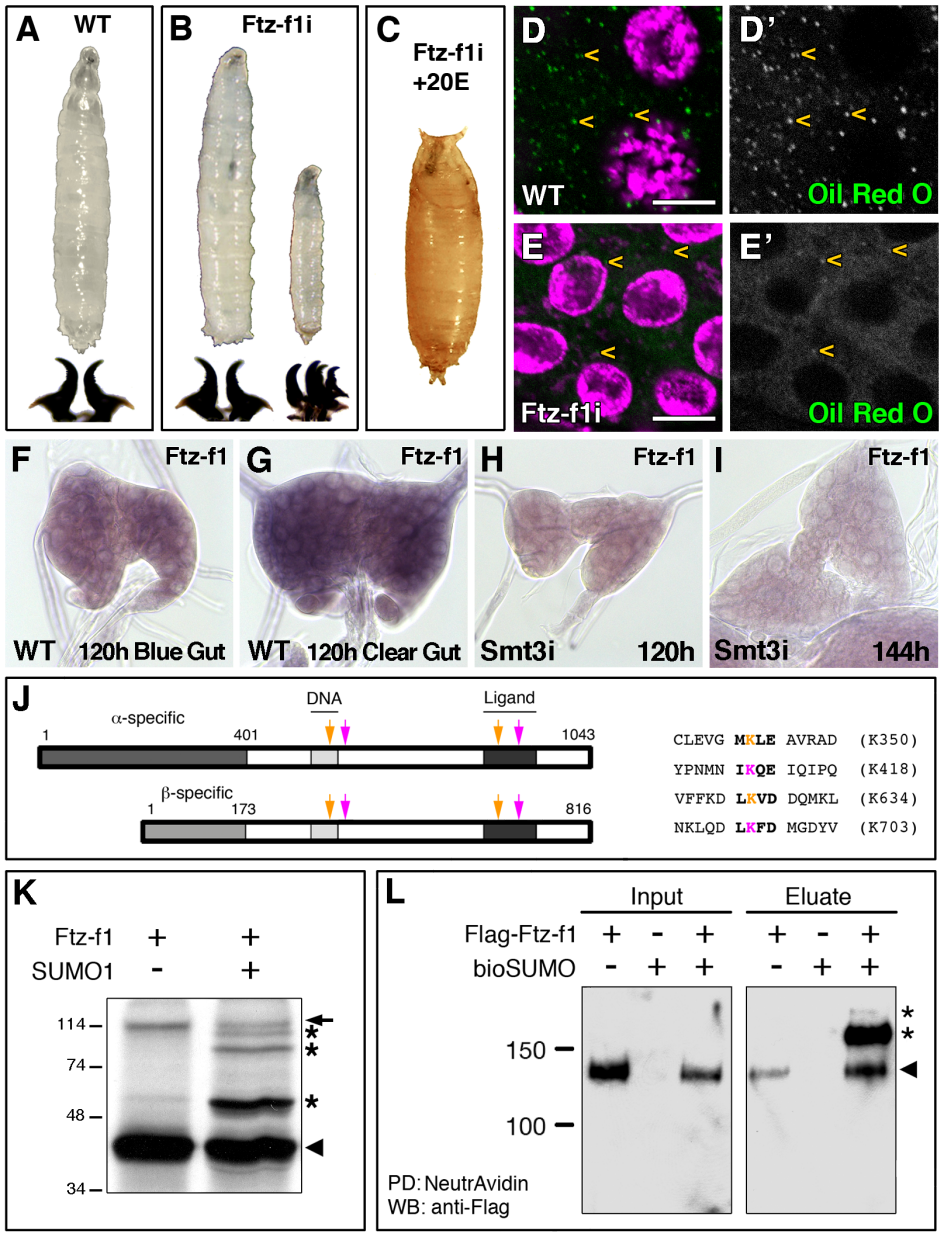
Ftz-f1 is required for pupariation and lipid uptake and is modified by SUMOylation *in vivo* and *in vitro*. (A) Wild type (WT) L3 larva and mouth hooks. (B) Most of the *phm-Gal4>UAS-ftz-f1 RNAi* (*ftz-f1i*) larvae arrested development at L3, while some of them stop at L2–L3 transition, as shown by the mouth hook morphology. (C) L3 *ftz-f1i* larvae fed with 20E were able to pupariate. (D, E) Single plane confocal micrographs showing nuclei marked with DAPI (purple) and lipid droplets, indicated by yellow arrowheads, stained with Oil Red O (green) in L3 control (D) and *ftz-f1i* PGs (E). (D′, E′) Single green channels are shown in black and white. L3 *ftz-f1i* PG cells contained reduced number of lipid droplets. All pictures were taken under the same intensity settings. Scale bars indicate 10 µm. (F–I) Micrographs of *ftz-f1* mRNA *in situ* hybridization in PGs from WT (F, G) or *phm-Gal4>UAS-smt3 RNAi* (*smt3i*) larvae (H, I) at the indicated hours AEL. *ftz-f1* expression increases in clear-gut respect to blue-gut larvae (G *versus* F, respectively). In *smt3i* larvae *ftz-f1* expression is downregulated (H, I). All the *in situ* reactions were stopped at the same time and pictures were taken at the same magnification. (J) Schematic representation of α- and βFtz-f1 isoforms indicating the predicted SUMOylation sites (arrows) and the sequences for the high-scoring sites found in Ftz-f1. Pink arrows indicate the SUMOylation sites conserved in insects and orange arrows the SUMOylation sites conserved from insects to vertebrate NR5A2. Pink and orange Ks indicate the lysines where SUMO attachment could take place. Numbering is based on the βFtz-f1 isoform. (K) *In vitro* SUMOylation assay. Incubation of the Ftz-f1 protein in the presence (+) of SUMO1 changes the motility of the protein (asterisks). Arrowhead indicates the unmodified protein and arrow indicates an unspecific band. Molecular weight markers are shown to the left. (L) *In vivo* SUMOylation assay. S2R+ cell extracts containing the indicated plasmids are shown. Arrowhead indicates the unmodified Ftz-f1 protein, while the asterisks indicate the high molecular weight SUMOylated forms. Input and eluate of the pull down reaction (PD) are shown. Note that the unmodified protein interacts with the NeutrAvidin beads (arrowhead).

In *Drosophila*, *ftz-f1* encodes for two protein isoforms with distinct temporal expression patterns: αFtz-f1 expressed early in embryogenesis and βFtz-f1 expressed later in embryogenesis and during larval, prepupal and early pupal stages [27], [28]. βFtz-f1 has previously been implicated in regulating ecdysteroid titers during post-embryonic development, specifically at the prepupa to pupa transition [26], [28]. To investigate whether Ftz-f1 contributes to the *smt3* knockdown phenotype (herein referred to as *smt3i*), we silenced both isoforms of *ftz-f1* in the PG using *UAS-ftz-f1i* lines and the *phm-Gal4* driver. The resulting larvae will be referred to as *ftz-f1i*. Reduced levels of *ftz-f1* led to arrested development at larval stages (Figure 1A, 1B). Similar to the *smt3i* phenotype, *ftz-f1i* larvae arrested at third instar (L3) before pupariation, as shown by the mouth hook morphology (Figure 1B). These larvae did not pupariate and survived as L3 for several weeks. In contrast to the *smt3i* phenotype, we also observed larvae that arrested development at the transition from the second instar (L2) to L3, as shown by the double mouth hooks. These larvae die at 120 hours after egg lying (AEL; Figure 1B). The two larval phenotypes could reflect the silencing of the two *ftz*-*f1* isoforms.

The developmental arrest at L3 suggested that *ftz-f1i* larvae were not able to synthesize normal levels of ecdysteroids at the onset of pupariation. Accordingly, and similarly to other low ecdysteroid mutants including the *smt3i* larvae, *ftz-f1i* larvae fed with exogenous 20E pupariated (Figure 1C).

*smt3i* PG cells show a reduction in lipid droplets and sterol levels, in addition to changes in steroidogenic enzymes and transcription factors [7]. *ftz-f1i* PGs also showed reduced levels of lipid droplets per cell (Figure 1D, 1E), as well as reduced levels of the steroidogenic enzyme Dib (data not shown). Taken together, these results show that Ftz-f1 is required in the PG at the end of L3 to acquire appropriate levels of cholesterol and to process it into ecdysone.

### Regulation of Ftz-f1 by SUMOylation

It was previously reported that Ftz-f1 protein is reduced in *smt3i* PGs [7], which could explain why *ftz-f1* silencing phenocopies SUMO downregulation. SUMO could be necessary for *ftz-f1* transcription, for Ftz-f1 protein modification, or both. To clarify this point we analyzed *ftz-f1* expression in PGs from 120 hours AEL blue-gut wandering larvae (i.e. 5–12 hours before pupariation), and 120 hours AEL clear-gut larvae (i.e. 1–6 hours before pupariation). *ftz-f1* transcription is upregulated in clear-gut larvae compared to blue-gut (Figure 1F, 1G). However, *ftz-f1* expression is lower in *smt3i* PGs and it does not get upregulated in older larvae (Figure 1H, 1I). This indicates that SUMO is involved in *ftz-f1* transcriptional regulation.

It has been reported that the mammalian homologues of Ftz-f1 are modified by SUMOylation [14], [18]–[21]. Therefore, we decided to test whether Ftz-f1 can be modified by SUMO. We determined the potential SUMOylation sites in Ftz-f1 using the SUMOplot analysis program and the Phosida Posttranslational Modification Database (Figure 1J). The two isoforms share the C-terminal region, but contain different N-terminal regions (Figure 1J). The *in silico* analysis showed that αFtz-f1 and βFtz-f1 share four SUMO consensus sequences, all of them conserved among insects, of which three have high prediction scores (Figure 1J and data not shown). The SUMOylation consensus sites are located in the DNA binding domain, in the hinge region and two of them in the ligand binding domain (Figure 1J and Figure S1). Those located in the DNA binding domain and in the ligand binding domain, are conserved between insects and NR5A2 (LRH-1) but not NR5A1 (SF-1; Figure 1J and Figure S1).

Using an *in vitro* SUMOylation assay, our results showed that Ftz-f1 protein is modified in the presence of SUMO, appearing as additional slow-migrating bands (Figure 1K). In order to analyze whether Ftz-f1 can also be SUMOylated *in vivo*, we developed a SUMOylation assay in cultured *Drosophila* cells. Smt3 was expressed as a fusion with a biotinylation-target peptide (bioSUMO, see Materials and Methods), along with Ftz-f1 and BirA enzyme. In this assay, biotinylated SUMO-conjugated proteins were bound to NeutrAvidin beads allowing for the specific isolation of SUMOylated material. Our results show that full-length αFtz-f1 is SUMOylated in *Drosophila* S2R+ cells (Figure 1L), with the estimated molecular weight for the main band (low asterisk) corresponding to the addition of one bioSUMO moiety. An additional but weaker band was observed at a higher molecular weight (upper asterisk in Figure 1L), suggesting the possibility that Ftz-f1 could also be modified by more than one bioSUMO moiety *in vivo*.

Taken together, these results show that SUMOylation regulates Ftz-f1 in two ways. On one hand, it is necessary for *ftz-f1* expression and, on the other hand, the protein Ftz-f1 is modified by SUMO *in vitro* and *in vivo*, suggesting that the post-translational modification o Ftz-f1 could potentially contribute to Ftz-f1 function in the PG.

### SR-BIs expression is regulated by Smt3 and Ftz-f1 in PG cells

**Figure 2:**
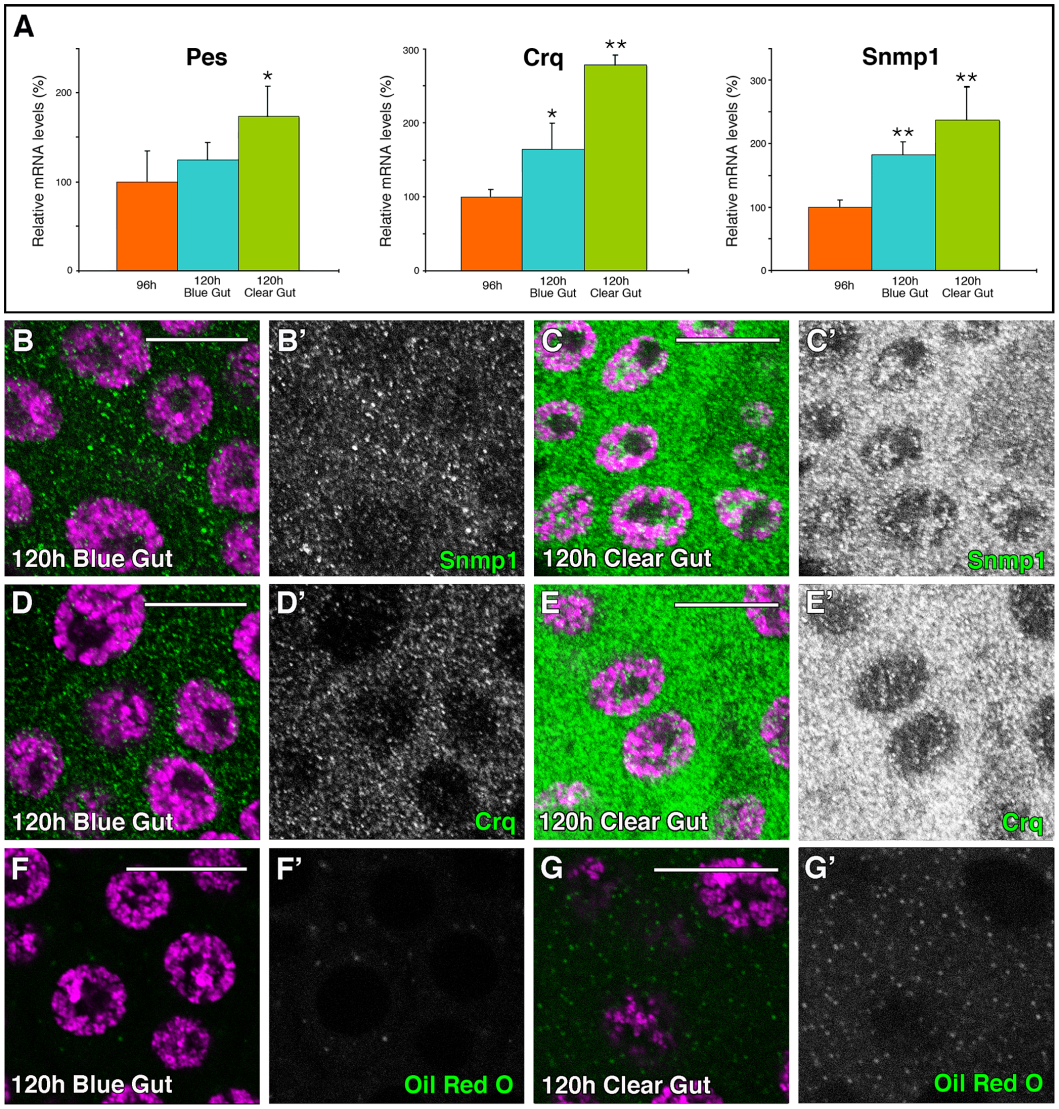
SR-BIs expression and lipid uptake is upregulated at late L3. (A) Graphical representation of the qPCR results showing the upregulation of the *Drosophila* SR-BI members *pes*, *crq* and *Snmp1* from 96 hours AEL to 120 hours AEL blue-gut and 120 hours AEL clear-gut larvae. Error bars indicate standard deviation. One asterisk (p<0.001) and two asterisks (p<0.0001) indicate significant upregulation respect to 96 hours larvae. (B–G) Single plane confocal micrographs taken under the same intensity settings. Nuclei are marked with DAPI (purple). (B–C) Expression of Snmp1 (green) is upregulated in WT PGs from 120 hours AEL blue-gut larvae (B) to 120 hours AEL clear-gut larvae (C). (D–E) Crq expression (green) is upregulated in WT PGs from 120 hours AEL blue-gut larvae (D) to 120 hours AEL clear-gut larvae (E). (F, G) The number of lipid droplets shows simultaneous increase in the PGs during the same time, as shown by Oil Red O staining (green). (B′–G′) Single green channel are shown in black and white. Scale bars indicate 20 µm.

The CD36 family of Scavenger Receptors is necessary for lipid uptake in various mammalian cell types [29]. We hypothesized that members of this family could be necessary for sterol uptake in the PG and could mediate some of the functions of Smt3 and/or Ftz-f1. Fourteen members of the CD36 family were identified in *Drosophila melanogaster* [30]. By *in situ* hybridization it was recently shown that three of these receptors, *peste* (*pes*), *croquemort* (*crq*) and *Snmp1* were upregulated in the PG at the onset of pupariation [31]. We confirmed this upregulation by quantitative real-time PCR (qPCR) of cDNA samples from precisely staged PGs at 96 hours AEL, 120 hours AEL blue-gut wandering larvae and 120 hours AEL clear-gut larvae (Figure 2A).

By immunostaining using specific antibodies we observed that Snmp1 was expressed in *Drosophila* PG cells at 120 hours AEL (Figure 2B, 2C). Interestingly, Snmp1 expression was upregulated from L3 blue-gut (Figure 2B) to L3 clear-gut larvae (Figure 2C). The same results were observed when antibodies against Crq were used (Figure 2D, 2E), suggesting a requirement for high levels of the Scavenger Receptors at the end of L3. The Scavengers expression upregulation from blue- to clear-gut larvae coincides with an increase in the content of lipid droplets of the clear-gut PGs (Figure 2F, 2G).

**Figure 3:**
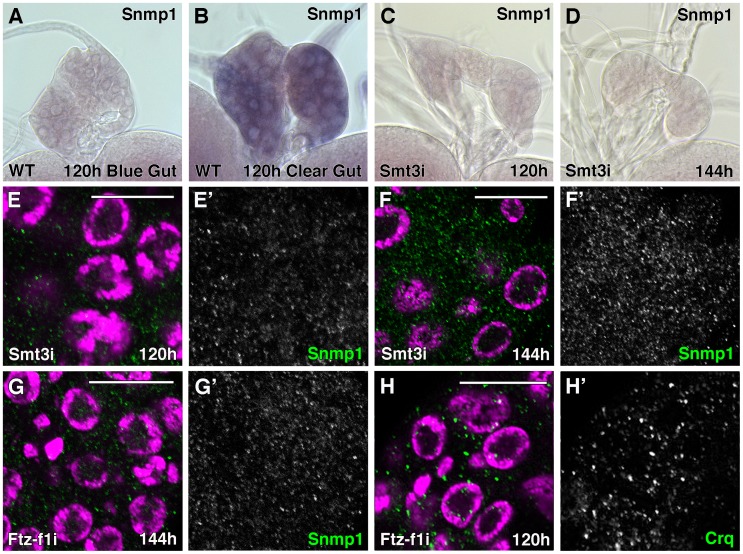
Expression of SR-BIs is reduced in *smt3i* and *ftz-f1i* PG cells. (A–D) Micrographs of *Snmp1* mRNA *in situ* hybridization in PGs from WT (A, B) or *phm-Gal4>UAS-smt3 RNAi* (*smt3i*) larvae (C, D) at the indicated hours AEL. *Snmp1* mRNA is upregulated in WT clear-gut larvae (B, compare with A). However, no expression is visible in *smt3i* PGs (C, D). All the *in situ* reactions were stopped at the same time and pictures were taken at the same magnification. (E–H) Single plane confocal micrographs taken under the same intensity settings. Nuclei are marked with DAPI (purple) and Snmp1 (E–G) or Crq (H) are shown in green. (E, F) *smt3i* PG cells have basal but reduced levels of Snmp1 at 120 hours AEL larvae (compare to WT in Figure 2B) but they do not upregulate SR-BIs expression at 120–144 hours AEL larvae. (G, H) *ftz-f1i* PG cells show Snmp1 (G) or Crq (H) expression highly reduced compared to Figure 2C or 2E, respectively. (E′–H′) Single green channels are presented in black and white. Scale bars indicate 20 µm.

As reported previously, the expression of Snmp1 is upregulated at the level of mRNA from blue- to clear-gut larvae (Figure 3A, 3B) [31]. Interestingly, we observed that the levels of expression of *Snmp1* are reduced, and are not upregulated, in *smt3i* PGs (Figure 3C, 3D). Similar results were obtained when specific antisense probe for *crq* was used (Figure S2A–S2D). However, *pes* expression is still present in smt3i PGs (Figure S2E–S2H), indicating different regulatory requirement for *pes* respect to *crq* and *Snmp1*.

At the level of proteins, we observed that Snmp1 expression was reduced in *smt3i* and in *ftz-f1i* PG cells (Figure 3E, 3F, 3G). Although basal levels of expression were evident in *smt3i* 120 hours larvae (Figure 3E), the upregulation of Snmp1 in *smt3i* PGs was never observed, even when we analyzed 144 hours AEL larvae (Figure 3F, compare to Figure 2C). The same results were observed when anti-Crq antibodies were used (Figure 3H, compare to Figure 2E).

These results indicate that SR-BIs are downstream of Smt3 and Ftz-f1 in the PG and suggest that SR-BIs might be required to mediate the role of Smt3 and Ftz-f1 in steroidogenesis during the larval to pupal transition.

### SR-BIs silencing in the PG phenocopies *smt3i* and *ftz-f1i* L3 developmental arrest

**Figure 4:**
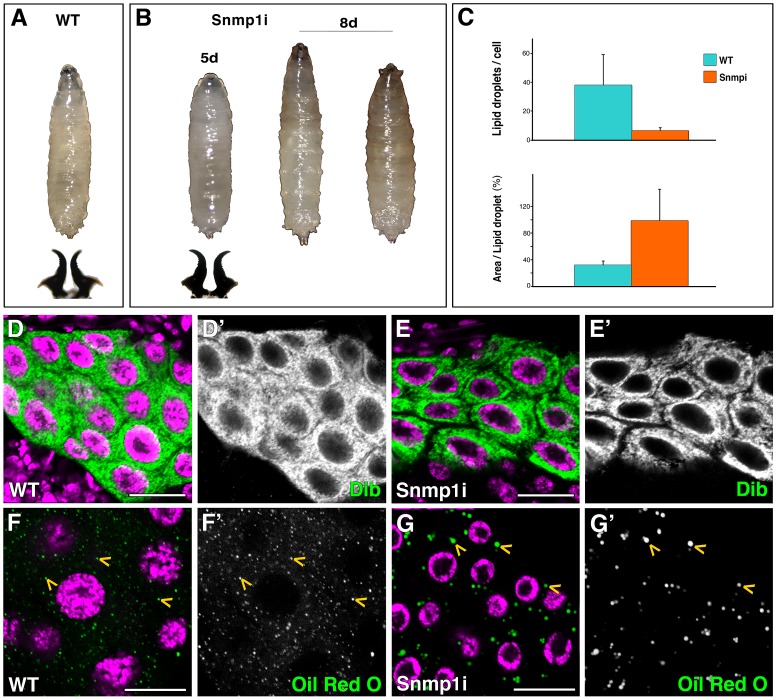
Snmp1 is required in the PG for pupariation and lipid uptake. (A) Wild type (WT) L3 larva and mouth hooks. (B) *phm-Gal4>UAS-Snmp1 RNAi* (*Snmp1i*) larvae arrested development at L3, as shown by morphology of mouth hooks, and were able to survive for several days. (C) Graphical representation of the average number of lipid droplets per cell and the area per lipid droplet in WT versus *Snmp1i* PG cells. (D–G) Single plane confocal micrographs taken under the same intensity settings. Nuclei are marked with DAPI (purple). (D, E) Dib expression is similar in WT (D) and *Snmp1i* PG cells (E). (F–G) Oil Red O staining shows the lipid droplets (yellow arrowheads) content in WT (F) and *Snmp1i* PG cells (G). Note that the number of lipid droplets was highly reduced, while the size of the lipid droplet was larger in *Snmp1i* compared to control PG cells. (D′–G′) Single green channels are shown in black and white. Scale bars indicate 20 µm.

To test the implication of the three *Drosophila* SR-BI members expressed in the PG in cholesterol uptake and steroidogenesis, we used the *phm-Gal4* line to silence *crq*, *pes* or *Snmp1* specifically in the PG. Interestingly, at 25°C *Snmp1* knockdown in the PG (herein called *Snmp1i*) led to L3 developmental arrest (Figure 4B). The *Snmp1i* L3 larvae survived for approximately ten days, darkened in the anterior part but failed to form a cuticle, the pseudo-pupae dying at this stage (Figure 4B). Unlike *smt3i* or *ftz-f1i*, the long-lived *Snmp1i* larvae had normal levels of expression of the cytochrome P450 enzyme Dib, indicating that the receptor does not have a major role in regulating this steroidogenic enzyme (Figure 4D, 4E). However, similarly to *smt3i* and *ftz-f1i*, *Snmp1i* PGs had a reduced content of lipid droplets, reaching between 5 to 16% of droplets per cell in comparison to controls (Figure 4C, 4F, 4G). Intriguingly, these droplets were abnormally big, being about three times bigger in *Snmp1i* cells compared to controls (Figure 4C, 4F, 4G). When *Snmp1i* L3 larvae were fed with exogenous 20E, 100% of the animals pupariated, and 66% led to adult flies (n = 30).

In contrast, knockdown of *crq* or *pes* in the PG at 25°C did not lead to any obvious phenotype. However, we observed L3 developmental arrest when silencing *pes* at 29°C (data not shown). Also in this case, the lipid droplets in the PG were less in number and larger in size than in controls (data not shown), which suggest that these receptors are involved in lipid uptake as well as in lipid droplet mobilization and/or the control of lipid droplets size.

### Smt3 is involved in lipid uptake in ovarian follicle cells

**Figure 5:**
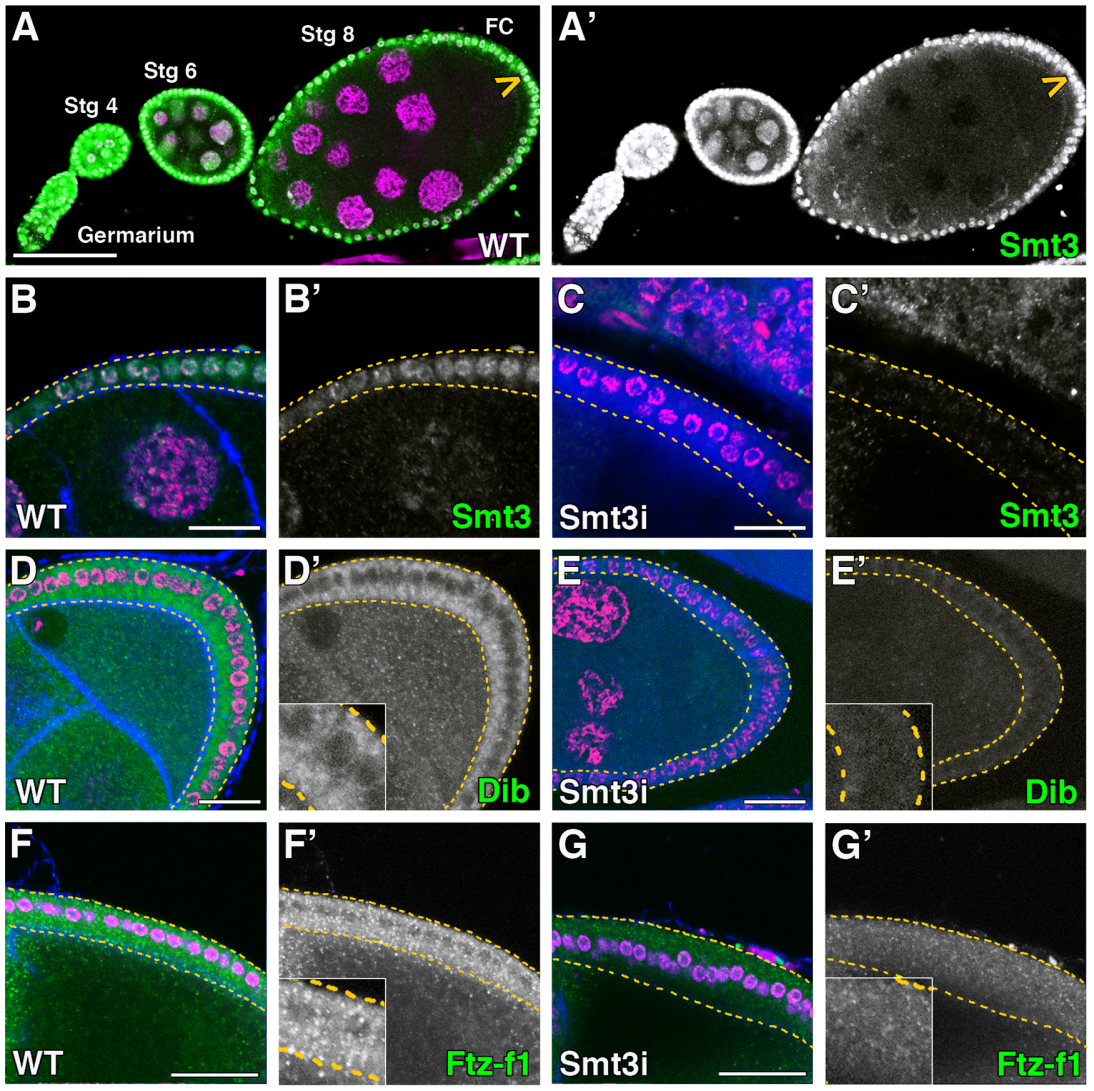
Smt3 is necessary for the expression of Dib and Ftz-f1 in follicle cells. (A–G) Single plane confocal micrographs were taken under the same intensity settings in *smt3* knockdown and control ovaries. Nuclei are labelled with DAPI (purple). F-actin cytoskeleton is shown in blue in B, D, F and G. GFP is shown in blue in C and E. Follicle cells are indicated by yellow dotted lines in B–G. (A′–G′) Single green channels are shown in black and white. Close-up of the follicle cells are shown in insets in D′–G′. Scale bars indicate 20 µm. (A) Expression of Smt3 (green) in the *Drosophila* germarium and egg chambers. Arrowhead points to follicle cells (FC). (B–C) Smt3 (green) expression levels in follicle cells in *T155-Gal4>UAS-GFP;UAS-smt3i* (Smt3i) (C) is severely reduced compared with the control (B). (D–G) Stage 8 and early stage 9 egg chambers showing in green the expression of Dib (D, E) or Ftz-f1 (F, G) in *T155-Gal4>UAS-GFP;UAS-smt3i* or *T155-Gal4>UAS-smt3i* (E, G) compared with the controls (D, F).

Besides PG, we wondered whether the molecular mechanism of lipid transport would be conserved in other steroidogenic tissues. The ovary is one of the sources of ecdysteroids in female adult insects. In cockroaches and locusts it has been shown that the follicle cells can synthesize and secrete ecdysone [32]–[34]. In *Drosophila*, *in vitro* synthesis and secretion of ecdysteroids has also been described in the ovary [35], [36]. *smt3* and other SUMOylation genes are expressed during oogenesis in *Drosophila* [37]. We examined Smt3 protein expression, which was localized mainly to the nucleus, and observed the highest levels in the germarium and in the follicle cells at stages 2–8 egg chambers, with weaker expression also evident in nurse cells at these stages (Figure 5A, 5B). At later stages of oogenesis, Smt3 expression in the follicle and nurse cells was maintained (data not shown). To ask whether the Smt3 requirement for sterol uptake is a common feature for steroidogenic tissues, we silenced *smt3* in the follicle cells using *UAS-smt3i* lines and the follicle-specific *T155*-Gal4 driver (Figure 5C) [38].

The ovary expresses several members of the cytochrome P450 enzyme family involved in ecdysteroid synthesis such as Phm, Dib, Shadow, Shade, Neverland and Dare [39]–[43]. We have focused our study from stage 8 until stage 10–11 of oogenesis, when the highest expression levels of these enzymes have been observed. In control female ovaries Dib expression started at stage 8 in the follicle cells, with the highest levels at stages 9–10, in correlation with the peak of ecdysone synthesis in the ovary (Figure 5D and data not shown) [44], [45]. Similarly to what happens in the PG [7], the expression levels of Dib in *smt3* knockdown follicle cells were drastically reduced (Figure 5E). In addition, Ftz-f1, which was expressed both in nurse and follicle cells, was also reduced in the *smt3* silenced follicle cells (Figure 5F, 5G), as shown in the PG [7].

**Figure 6:**
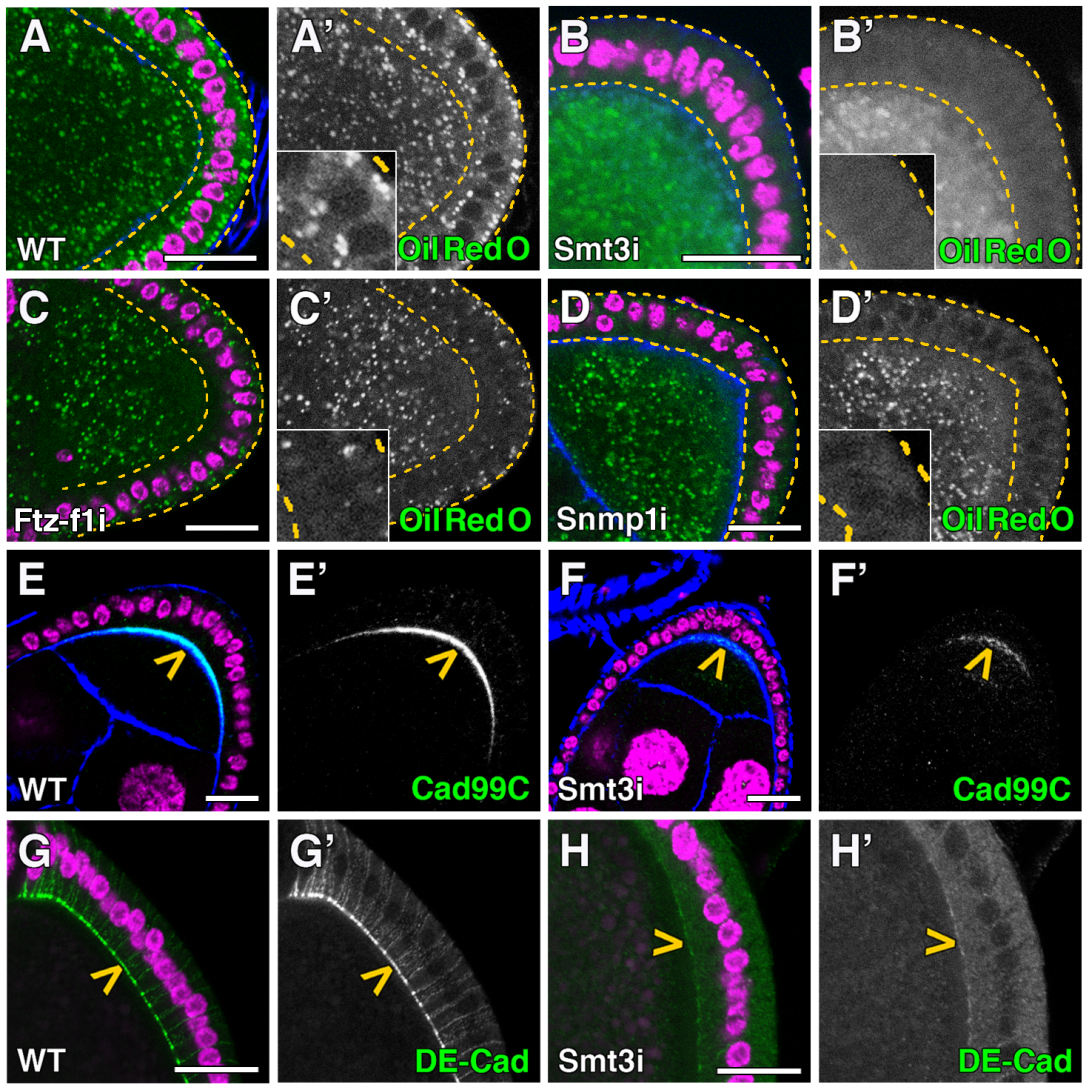
Smt3 and Snmp1 are necessary for lipid uptake in follicle cells. (A–H) Single plane confocal micrographs taken under the same intensity settings. Nuclei are labelled with DAPI (purple). F-actin cytoskeleton is shown in blue. Follicle cells are indicated by yellow dotted lines. (A′–H′) Single green channels are shown in black and white. Close-up of the follicle cells are shown in insets in A′–D′. Scale bars indicate 20 µm. (A–D) Lipid droplets shown by Oil Red O staining (green) in follicle cells of WT (A), *T155-Gal4>UAS-smt3i* (B), *T155-Gal4>UAS-ftz-f1i* (C) or *T155-Gal4>UAS-Snmp1i* (D) adults. (E, F) Cad99C (green; yellow arrowheads) is very much reduced in *T155-Gal4>UAS-smt3i* (F) respect to WT (E). (G, H) DE-Cad (green; yellow arrowheads) is also reduced in *T155-Gal4>UAS-smt3i* (H) respect to WT (G).

At the stages analyzed, the follicle and nurse cells and the oocyte show high number of lipid droplets (Figure 6A). Interestingly, we observed a strong reduction in the number of lipid droplets in *smt3i* follicle cells, which correlate with the observations shown in the PG (Figure 6B and data not shown). However, the oocyte was not completely depleted of lipids as it received lipids from the nurse cells throughout the ring canals (Figure S3A), which might explain why these oocytes are able to give rise to viable embryos. The reduction of Ftz-f1 levels in the follicle cells produced a more severe phenotype with death of many ovarioles. However, those that survived showed also a reduction in the number of lipid droplets in the follicle cells, this reduction being not as strong as in the *smt3i* phenotype (Figure 6C).

To follow up our observations in the PG, we analyzed whether the SR-BI family could be involved in the lipid uptake in the ovary. Indeed, we observed that Snmp1 knockdown in the follicle cells clearly reduced the lipid droplet content in these cells (Figure 6D). Interestingly, the few droplets observed in some cells were located in the basal part, suggesting a mobilization impairment of droplets from the basal to the apical surface of follicle cells (Figure S3B).

During the stages 8–10 of oogenesis, follicle cells accumulate over the oocyte and become columnar, showing numerous microvilli on their apical surface [46]. These microvilli can be detected by the expression of Cad99C, a cadherin involved in the regulation of the microvilli length [47], [48]. We detected Cad99C protein on the apical plasma membrane of the follicle cells surrounding the oocyte (Figure 6E). However, in *smt3i* follicle cells Cad99C expression was highly reduced, which suggests a microvilli malformation in these cells (Figure 6F). DE-Cad, which marks adherent junctions and is required for centripetal cell migration, was also greatly reduced in *smt3i* follicle cells (Figure 6G, 6H) [49]. We observed a slight delay in the centripetal migration of follicle cells and very small gaps in the vitelline membrane that might be attributed to the reduction in the expression level of cadherins (data not shown). Even with these changes, egg fertility was only slightly affected and viable embryos were obtained.

These results show that reduced levels of Smt3 in follicle cells affect the levels of Dib and Ftz-f1, alter lipid droplet size and distribution, and affect membrane surface area (in this case, by altering microvilli), suggesting that Smt3 performs parallel functions in the PG and in the ovary. Moreover, Snmp1 could play similar roles in lipid uptake and mobilization in the PG and in the follicle cells.

### Snmp1 restores the lipid droplet content of *smt3* knockdown in PG and follicle cells

**Figure 7:**
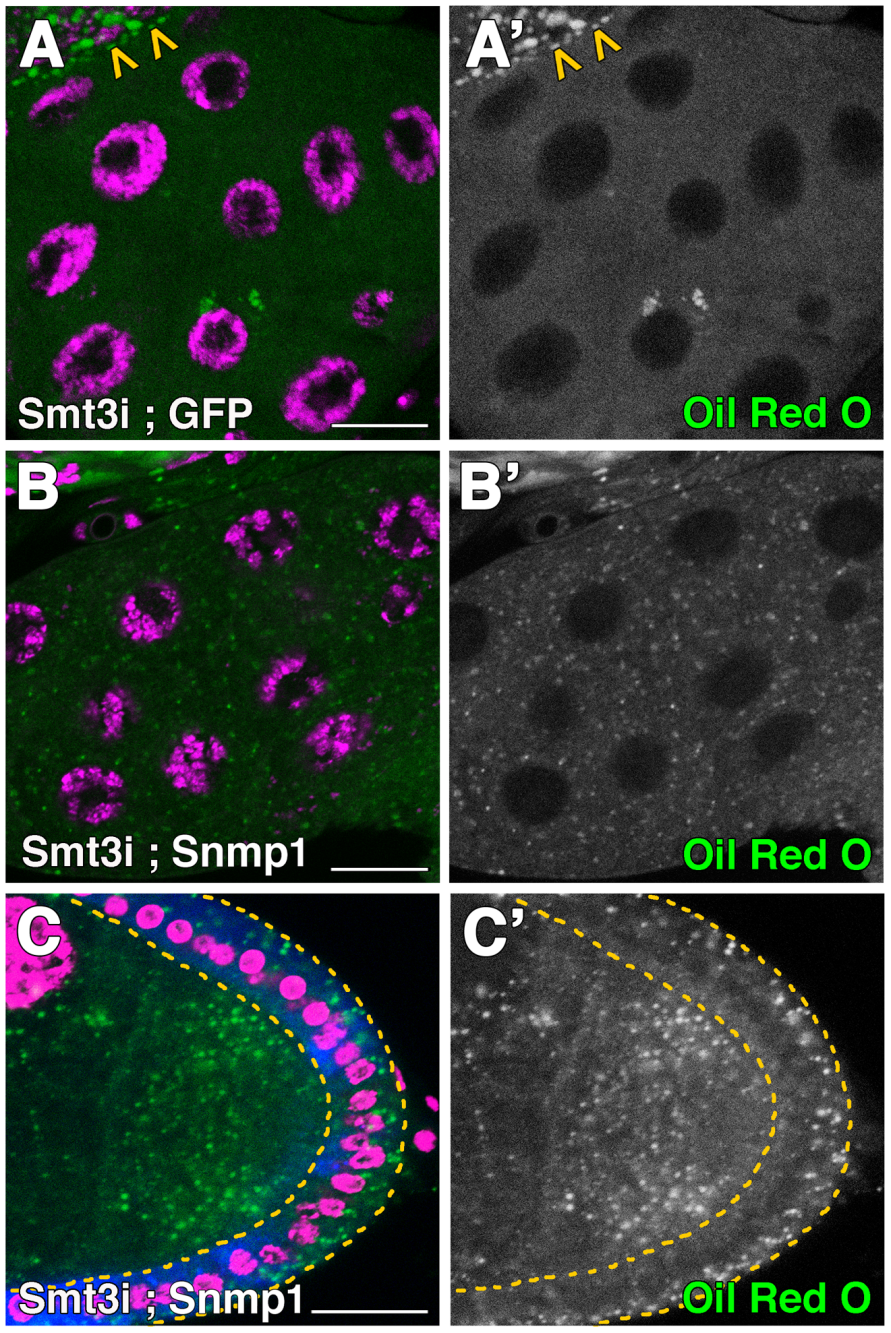
Snmp1 restores the lipid droplets content in *smt3i* cells. (A–B′) Single plane confocal micrographs taken under the same intensity settings showing Oil Red O staining (green) in PGs of *phm-Gal4>UAS-GFP;UAS-smt3i* (A) or *phm-Gal4>UAS-Snmp1;UAS-smt3i* (B). Arrowheads in A indicate the brain hemisphere with normal levels of lipid droplets, as *smt3* was not silenced there. (C) Confocal micrograph showing Oil Red O staining (green) of follicle cells *T155-Gal4>UAS-Snmp1;UAS-smt3i*. GFP is shown in blue. Follicle cells are indicated by yellow dotted lines. (A′–C′) Single green channels are shown in black and white. Nuclei are labelled with DAPI (purple). Scale bars indicate 20 µm.

Our results showed that silencing *Snmp1* phenocopies the impairment of lipid uptake of *smt3* or *ftz-f1* knockdowns. To test the role of *Snmp1* in the *smt3i* phenotype in the PG, we over-expressed *Snmp1* in an *smt3i* background. Interestingly, we observed the rescue of the number of lipid droplets per cell in 51% of the larvae (n = 29; Figure 7A, 7B). The lipid droplets were comparable to the controls in size, indicating that the overexpression of *Snmp1* was able to restore the uptake of lipids and their mobilization in the PG. Furthermore, Snmp1 was able to rescue the lipid droplets content in *smt3i* follicle cells (Figure 7C). These results demonstrate the role of Snmp1 in sterol uptake in both the PG and the ovary and underline the relevance of the *Drosophila* SR-BI family in steroidogenesis.

### SUMOylation of Ftz-f1 modulates *Snmp1* expression

**Figure 8:**
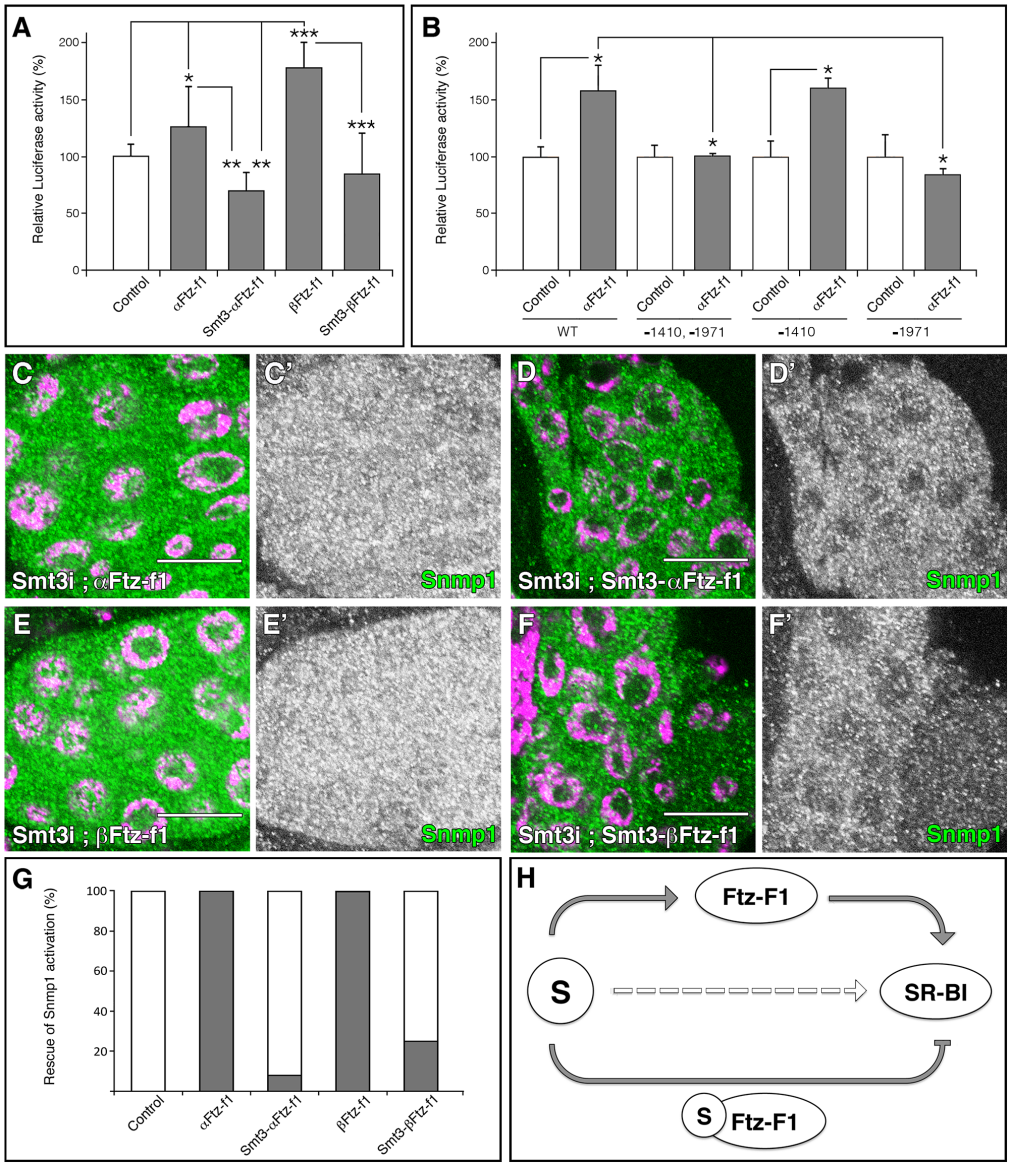
SUMOylation of Ftz-f1 modulates Snmp1 expression. (A, B) Graphical representations of the luciferase activity from WT *Snmp1-Luc* (A) or the indicated mutant vectors (B) cotransfected in S2R+ *Drosophila* cells with plasmids expressing the indicated proteins. As control, empty *pUASTattB* vector was used. A single asterisk indicates p<0.05, two asterisks p<0.001 and three asterisks p<10^−6^. (C–F) Single plane confocal micrographs taken under the same intensity settings showing Snmp1 staining (green) in PGs of *phm-Gal4>UAS-smt3i;UAS-αFtz-f1* (C), *phm-Gal4>UAS-smt3i;UAS-Smt3-αFtz-f1* (D), *phm-Gal4>UAS-smt3i;UAS-βFtz-f1* (E) or *phm-Gal4>UAS-smt3i;UAS-Smt3-βFtz-f1* (F). Nuclei are labelled with DAPI (purple). (C′–F′) Single green channels are shown in black and white. Scale bars indicate 20 µm. (G) Graphical representation of the proportion of PGs that show Snmp1 expression in an *smt3i* background (grey bars). White bars indicate no Snmp1 expression rescue. The transgenes used for the rescue experiments are indicated. (H) Schematic summary of the results, where S means Smt3. Grey arrows indicate the requirement of SUMOylation for Ftz-f1 and SR-BI expression, and the regulation of SR-BI by Ftz-f1 or Smt3-Ftz-f1. Broken open arrow indicates hypothesized posttranslational modification of Scavengers by Smt3.

Our results suggest that SR-BIs are downstream of Ftz-f1. To study whether *Snmp1* transcription is mediated, at least in part, by Ftz-f1 we analyzed its promoter region. Interestingly, the *Snmp1* locus contains two putative Ftz-f1 binding sites (TCAAGGTgG, position −1410 from the initial methionine; CCAAGGgCA, position +1666) that only differ in one nucleotide from the consensus sequence 5′-PyCAAGGPyCPu-3′. In addition, an atypical SF-1 binding site (TttGGGCCA, position −1974) that contains the core of the consensus sequence TCAGGGCCA [50], is present in the promoter and could also be implicated in *Snmp1* regulation. We examined whether Ftz-f1 could activate *Snmp1* transcription using a reporter gene cloned downstream of a 2 Kb fragment located at 5′ of the *Snmp1* transcription initiation point. Interestingly, α and βFtz-f1 activated luciferase activity significantly in S2R+ *Drosophila* cells (Figure 8A). A mutation in position −1971, which eliminates the atypical SF-1 binding site reduces luciferase activity in presence of αFtz-f1 (Figure 8B), suggesting that activation depends of Ftz-f1 binding to that site.

Fusions of SUMO with the protein of interest can be used as a model of constitutive SUMOylation without the pleiotropic effects of overexpressing SUMO in the cells [51]. In addition, fusions of Ubc9, the E2 SUMO conjugating enzyme, are also used successfully as model for constitutive SUMOylation [52]. To examine the effect of Ftz-f1 modification on *Snmp1* activation, we analyzed the transcriptional activity of Smt3-βFtz-f1 and Smt3-αFtz-f1 fusion proteins (Figure 8A). Cotransfection assays showed that while α or βFtz-f1 were able to increase the luciferase activity compared with the control vector, Smt3-αFtz-f1 or Smt3-βFtz-f1 caused a reduction in the level of transcription (Figure 8A). This effect was reproduced using the Ftz-f1 proteins fused to Lesswright, the *Drosophila* homologue to Ubc9 (data not shown). These results suggest that SUMOylation reduces Ftz-f1 activation capacity on *Snmp1*.

According to our previous data, *ftz*-*f1* expression depends on SUMO. In turn, *Snmp1* expression depends of Ftz-f1. In fact, α or βFtz-f1 overexpression in the PG rescues Snmp1 expression in an *smt3i* background in 100% of the PGs analyzed (n = 37 and n = 36, respectively; Figure 8C, 8E, 8G, compare with Figure 3E). We then examined *in vivo* the differences of activity between α or βFtz-f1 and Smt3-α or Smt3-βFtz-f1. Smt3–αFtz-f1 or Smt3–βFtz-f1 overexpression only recovered Snmp1 expression in 8 or 25% of the PGs, respectively (n = 25 and n = 39; Figure 8D, 8F, 8G), suggesting a difference in the activity of Ftz-f1 depending on its SUMOylation status.

## Discussion

Here, we have investigated the effect of SUMOylation on SR-BI and Ftz-f1 activities in the context of steroidogenesis. We show that SUMOylation has a dual role on Ftz-f1 function. On one hand, *ftz-f1* transcription depends on SUMO. On the other hand, SUMO modifies Ftz-f1, reducing its capacity to activate Snmp1 transcription. In addition, we demonstrated that *Drosophila* SR-BI family is involved in steroidogenesis by regulating the cholesterol uptake in the PG required for the synthesis of ecdysone. Our results also show that Snmp1 is involved in cholesterol uptake, acting downstream of SUMO and Ftz-f1, and is able to recover the lipid content in *smt3i* PGs. Furthermore, we showed that SUMO and the Scavenger Receptors are also involved in lipid capture and mobilization in the ovarian follicle cells.

### SUMOylation in steroidogenesis

SUMO modification, a highly conserved pathway throughout evolution, is known to impact the activity, interactions, localization and stability of proteins [53]. A number of studies during the last years have clearly established the essential role of SUMOylation during development. In *Drosophila melanogaster*, the single SUMO protein Smt3 is expressed during development and is highly enriched during embryogenesis and in adult females [54]–[56]. At later stages it is predominantly expressed in the central nervous system and in the gonads [37], [56], [57]. Components of the *Drosophila* SUMO conjugation pathway have also been implicated in diverse processes such as embryogenesis, wing morphogenesis, central nervous system development, neurodegeneration, photoreceptor development and immune response (reviewed in [58], [59]. In addition, Smt3 is required in the PG for the correct function of the ecdysteroid biosynthetic pathway at the time of puparium formation, which derives from defects in cholesterol uptake and formation of intracellular channels [7]. We have now shown that Smt3 requirement for lipid uptake is a common feature for steroidogenic tissues by analyzing its function in the ovary, a tissue that also requires cholesterol for the synthesis of ecdysone. In addition, we show that SUMO is required to activate *ftz-f1* transcription. During the late larval pulse of ecdysone, the transcription factors E75, DHR3 and the Nitric Oxide Synthase (NOS) are known to regulate Ftz-f1 expression. Ftz-f1 is a direct target of DHR3 [60], [61] and E75 suppresses this DHR3 mediated expression of βFtz-f1 [62]. NO, produced by NOS, prevents the E75 function as suppressor of DHR3 [63]. Therefore, these factors could be responsible for the SUMO dependent *ftz-f1* transcriptional activation. Noteworthy, NOS is modified by SUMO [64], [65] and its downregulation in the PG prevents pupariation [63]. An interesting question to be analyzed in the future will be the study of the biological role of NOS SUMOylation in the regulation of *ftz-f1* expression.

Although several *Drosophila* Smt3-modified proteins have been identified, the effect of SUMOylation remains unknown for most of them. A proteomic study in early *Drosophila* embryos identified 140 SUMOylation targets that confirmed the role of this pathway in Ras signaling, cell cycle and pattern formation [66]. Smt3 also regulates negatively JNK signaling through sequestering HipK in the nucleus [67]. Further identification of SUMO targets at different developmental stages will be particularly important. We are especially interested in PG proteins modified by SUMO during the larval-pupal transition since Smt3 function is required in this crucial developmental window. In that context, the SUMO modification of *Drosophila* Ftz-f1 described here, to our knowledge, for the first time is particularly exciting. Ftz-f1 hypomorphic regulatory mutants show defects at the prepupal-pupal transition, such as failure of head eversion and salivary gland cell death, and suggest a function for this transcription factor in muscle contraction events at this transition [28], [68], [69]. βFtz-f1 expression in late first and second instar larvae and its role in molting has also been described in *Drosophila* and other insects [28], [70], [71]. However, the precise expression pattern and the role of Ftz-f1 in *Drosophila* PGs from third instar larvae were not completely understood. Our results show that disruption of Ftz-f1 in the PG by RNAi impairs development at late L3, which clearly proves that Ftz-f1 is required at the larval-pupal transition. Interestingly, Ftz-f1 knockdown in the PG results not only in reduction of Dib expression as expected, but also in diminution of Snmp1 expression and a significant decrease of lipid levels. Therefore, our study implicates Ftz-f1 in sterol homeostasis in the PG as well as in the ovary, suggesting that this role could be extended to more steroidogenic tissues. Interestingly, Nhr-25, the only *C. elegans* member of the NR5A family, controls the larval to adult transition by regulating an endocrine program of lipid uptake and synthesis [72], [73].

A growing number of nuclear receptors are also known to be targets of SUMOylation (reviewed in [74], [75]. In mammals, the nuclear receptor coregulator KLF5 (Krüppel-like transcription factor 5) uses SUMOylation as a molecular switch to repress or activate genes involved in lipid catabolism [76]. Other transcription factors modified by SUMO and involved in energy metabolism include PPAR-γ, C/EBPs and SREBPs [77]–[79]. In addition, a deSUMOylating enzyme, SeNP2 plays a critical role in the control of adipogenesis [80]. SUMOylation of SF-1, as suggested for other transcription factors, attenuates its transcriptional activity [18]. However, only a subset of SUMO sensitive targets seems to be affected [81]. On the other hand, Androgen receptor interacting protein 4, which interacts with SUMOylated SF-1, suppresses SF-1 mediated transcription [82]. Recently, the elimination of SF-1 SUMOylation in mice has been described [21]. UnSUMOylable SF-1 mutants activated Sonic hedgehog signaling and altered other potential SUMO sensitive targets, leading to endocrine abnormalities and changes in cell fate. SUMO modification has also been associated with increased transcriptional activity of nuclear receptors, as reported for retinoid acid receptor related orphan receptor α (ROR α) and estrogen receptor α (ERα). Interestingly, SUMOylation at the hinge region of both nuclear receptors has been associated to transcriptional activation [83], [84]. SUMOylation of Ftz-f1, as occurs with its orthologue SF-1, could be an important mechanism to control its activity and probably a correct ratio of SUMOylated to unmodified Ftz-f1 must be maintained for proper development. As for SF-1, SUMOylation of Ftz-f1 seems to reduce its capacity of transcriptional activation on Snmp1. What could be the biological function of Ftz-f1 SUMOylation? One possibility might be that SUMOylation attenuates Ftz-f1 function after pupariation. As the first peak of ecdysone production subsides, perhaps Ftz-f1 SUMOylation and reduced levels of SR-BI contribute to this downregulation, separating it from the second ecdysone peak that drives pupation itself (10–12 hours after puparium formation).

### *Drosophila* SR-BIs requirement for cholesterol uptake and mobilization in the steroidogenic tissues PG and ovary

Cholesterol, a main component of the cell membranes, is also important for the synthesis of steroid hormones. Steroid hormone biosynthesis requires, in addition to correct cholesterol uptake, appropriate intercellular and intracellular transport. Insects, which are incapable of synthesizing cholesterol, incorporate it from the diet through intestinal absorption and then transport it to different tissues via open circulation in the hemolymph associated with the lipoprotein lipophorin [85]. Several ultrastructural changes have been described in active PG cells, such as increased agranular ER, mitochondria and increased intracellular channels and nuclear folding that correlate with the sterol uptake and/or release of ecdysone [7], [86]–[88]. However, the mechanisms used to incorporate cholesterol in the PG and secrete ecdysone are still largely unknown.

Two main pathways have been described for cellular uptake of lipoprotein-cholesteryl esters: the low-density lipoprotein (LDL) receptor mediated endocytic uptake of LDL-cholesterol and the “selective” cholesteryl ester uptake pathway mediated by SR-BI. In insects, proteins related to the mammalian LDLR, lipophorin receptors, have been identified [89]. Recently, the function of *Drosophila* Lpr1 and Lpr2 for neutral lipid uptake in imaginal disc cells and oocytes have been described; however, the phenotype of *lpr1* and *lpr2* mutants does not suggest a role for these receptors in the PG or in the larval-pupal transition [89].

In mammalian steroidogenic tissues the SR-BI “selective pathway”, without endocytic uptake, seems to be the main one used to satisfy the cholesterol requirements for hormone synthesis. [90]–[92]. Interestingly, SR-BI is also necessary for the formation of the microvillar channels of the adrenal gland, as shown by the reduction of channels in SR-BI null mice and the increased formation of these channels after overexpression of SR-BI [24], [93]–[96]. SR-BI is also expressed in the rat ovary where, similar to adrenocortical cells [95], it is detected on microvilli and membranes of microvillar channels that contained trapped lipoproteins [97]. The expression of SR-BI is regulated by the nuclear receptors SF-1 and LRH-1, supporting the significance of these receptors in lipid capture for steroidogenesis [22], [23], [98]. Other factors such as the hormones ACTH, estrogen or gonadotropin induce SR-BI expression [99], [100].

The *Drosophila* CD36 gene family consists of 14 genes [30], [101]. Recently, the expression of Snmp1, Crq and Peste in steroidogenic tissues such as PG, ovaries and testes was described [31], pointing to a role for these receptors in these tissues. Significantly, the expression of these receptors in the PG was upregulated at the moment of pupariation when high levels of ecdysteroids are required [31] and this work). Silencing *Snmp1* or *pes* in the PG produced the developmental arrest at L3 prior to pupariation. These results indicate that these receptors are not functionally equivalent and the presence of one of them cannot substitute for the other in the PG. Alternatively, reduction of the levels of one of the receptors might be enough to lower the total content of Scavengers and, therefore, the total capacity of the cell for capturing lipids. Interestingly, overexpression of *Snmp1* is able to rescue the lipid content of *smt3i* PG cells. However, this rescue of lipid content is not sufficient to allow the larval-pupal transition, suggesting that the cells are still unable to produce a threshold level of ecdysone. There could be several explanations for this. For instance, overexpression of one of the receptors would be enough for lipid capture but not for lipid mobilization. In this respect, abnormally large lipids droplets were observed in *Snmp1i* PG cells, which suggest an additional role in sterol mobilization or a function in the regulation of the lipid droplet size. Several proteins have been shown to affect the size of the lipid droplets such as Rab small GTPases, sterol regulatory element binding protein cleavage activating protein (SCAP) and isoforms of phosphocholine cytidylyltransferase [102], [103]. Mutants for other proteins that promote intracellular transport of lipids in the PG, such *npc1a* mutants, have abnormal accumulation of intracellular sterol and are unable to molt due to low levels of ecdysone [104]–[106].

We showed that the expression of SR-BIs increases at the onset of pupariation, coinciding with an increase in PG's lipid content. Moreover, our results showed that SR-BIs are regulated by Ftz-f1. Interestingly, the *Snmp1* locus contains two putative Ftz-f1 binding sites and an atypical SF-1 binding site that could be implicated in Snmp1 regulation. Indeed, experiments in cultured cells and *in vivo* showed that Ftz-f1 is able to activate *Snmp1* promoter. *Snmp1* might not be the only Scavenger Receptor regulated by Ftz-f1 in the PG. Furthermore, in addition to SUMOylation influencing the capacity of Ftz-f1 to regulate SR-BIs expression (Figure 8H), we observed that Snmp1 contains two high score putative SUMOylation sites (data not shown). Is Snmp1 modified by SUMO and could this modification affect its function in cholesterol uptake/transport during the larval to pupa transition? Does SUMOylated Ftz-f1 affect the regulation of other SR-BIs in clear-gut larvae? We cannot discard the possibility that other proteins involved in ecdysone synthesis or transport are SUMOylated. The fact that viability is not rescued by Snmp1 overexpression, suggests that this is indeed the case. These questions remain unanswered and will be addressed in the future.

In summary, we demonstrated that *Drosophila* SR-BI family and Ftz-f1 participate in steroidogenesis downstream of SUMOylation by regulating the lipid uptake in the PG required for the synthesis of ecdysone. The participation of these factors in lipid uptake is conserved in other steroidogenic tissues, suggesting a general role for SUMO, Ftz-f1 and SR-BI in lipid uptake. Our data provide new insight into the lipid homeostasis of the organism.

## Materials and Methods

### Drosophila strains

Flies were raised on standard *Drosophila* medium at 25°C. The wild-type (WT) control strain was Vallecas. Gal4 strains were *phm-Gal4,UAS-mCD8::GFP/TM6B,Tb* (called *phm-Gal4*, obtained from P. Leopold and C. Mirth) [107], [108] and *P(GawB)T155-Gal4* (Bloomington *Drosophila* Stock Center- BDSC). UAS-RNAi lines were: *UAS-smt3i* [7]; *UAS-ftz-f1i* (Vienna *Drosophila* RNAi Center- VDRC- #2959, which recognizes *α* and *βftz-f1* isoforms); *UAS-pesi* (VDRC #33155); *UAS-crqi* (VDRC #45883); *UAS-Snmp1i* (VDRC #04210) and *UAS-Snmp1i* (NIG-FLY #7000R-3). Overexpression UAS lines used were obtained from BDSC: *y^1^w^67c23^;P(EPgy2)crq^EY14489^* (#20939); *w;P(UAS-Snmp1.B)217.1/CyO;TM2/TM6B,Tb^1^* (#25044); w^*^;P(UAS-Snmp1.YFP(2)273.4/CyO;TM2/TM6B,Tb^1^ (#25046) and w^*^;P(UAS-Snmp1-EGFP)218.3/CyO;TM2/TM6B,Tb^1^ (#25045). Information about other strains can be found in FlyBase (http://flybase.bio.indiana.edu).

### Plasmid construction and generation of transgenic strains

Full length *α* and *βftz-f1* cDNA sequences (EMBL database accession numbers HE716957 and HE716956, respectively; GeneArt) were cloned into the *EcoR*I-*Xba*I sites of *pUASTattb* vector [109] to generate *pUASTattb-αftz-f1* and *pUASTattb-βftz-f1*, respectively. 3×Flag sequences were inserted into the *Eco*RI-*Bgl*II sites. To generate constitutively SUMOylated forms, degenerated nucleotide *smt3* sequence that encodes for WT Smt3 protein (EMBL database accession number FN539078) [110] was introduced into the *Asc*I and *Pac*I sites of the previous vectors to generate *pUASTattb-Smt3-αftz-f1* and *pUASTattb-Smt3-βftz-f1*, respectively.

Renilla and firefly luciferase (Fluc) were amplified from psiCHECK2 (Promega) by PCR and exchanged for GFP in *Ac5-STABLE1* (*Eco*RI-*Hind*III) [111] to generate *Ac5-FFluc-STABLE1*. For the construction of *pSnmp1-FLuc* genomic DNA was used as template to amplify a PCR fragment containing 2 Kb of the *Snmp1* upstream region (including 77 bp of 5′UTR) using the oligonucleotides Snmp1(−2000) (5′- GATCAGATCTTGAGCACTTAGGCATTTTCAAAACTATTTGGG -3′) and Snmp1(+1) (5′- GATCGAATTCCTCTGGGCAATGTTTCGATCTCTACTC -3′) (numbering based on initiator methionine). The resulting *Bgl*II-*Eco*RI fragment was used to replace the Actin5C promoter in *Ac5-FFluc-STABLE1*. Two potential Ftz-f1 binding sites were identified based on published consensus sites. Fragments were prepared for individual and double mutants in these potential Ftz-f1 binding sites using 2-step overlap extension PCR, using the forward and reverse primers Ftz-f1Mut(−1971) (5′- CTTAGGCATTTTCAAAACTATTTG**tt**CCAGCAATAATTGGTAGCAAAC -3′) and Ftz-f1Mut(−1410) (5′-GAGCCCAGTTAGCCGGTCAA**tt**TGGCAGAGCATCTAACTTAAATGG -3′) (mutated nucleotides in lowercase and bold). Resulting fragments were used to replace *Snmp1* WT promoter sequence to generate *pSnmp1Mut-FLuc* plasmids. All constructs were fully sequenced.

To generate *pUASTattb-crq* and *pUASTattB-pes*, ESTs RE02070 and RE21078 inserted in the PFLC1 plasmid (*Drosophila* Genomics Resource Center- DGRC), were digested with *Eco*RI and *Bam*HI, or *Mfe*I and *Bgl*II, respectively. Fragments were inserted in *pUASTattb* [109] digested with *Eco*RI and *Bgl*II. Transgenic lines were generated following standard transformation procedures [112].

### *In vivo* and *in vitro* SUMOylation assays

SUMOylation motifs were identified using SUMOplot (http://www.abgent.com/sumoplot) software and Phosida Posttranslational Modification Database (http://www.phosida.com). For the *in vitro* procedure *ftz-f1 cDNA* (LD34889, DGRC) was translated using TNT-T7 (wheat germ extract; Promega), to which 35S-methionine was added (Amersham Biosciences and Pierce). This cDNA construct contains three out of the four conserved SUMOylation consensus sites. Translated Ftz-f1 was incubated with an ATP-regenerating system, SUMO-1, Ubc9 and E1 activating enzyme (Biomol). Reactions were incubated at 30°C for 2 h, resolved by SDS-PAGE and exposed.

For cellular SUMOylation assays we developed new technology based on Franco et al. [113]. In brief, a plasmid encoding a form of Smt3, capable of being biotinylated, as well as the enzyme necessary for biotinylation, BirA, were introduced into cells. Any proteins that undergo SUMOylation will also be biotinylated facilitating their recovery using streptavidin beads. BirA was amplified from the *UAS-(bioUb)6-birA* vector [113] and cloned in *pAc5-STABLE2-Neo* [111] by substituting GFP, generating *pAc5-FLAGmCherry-BirA*. To generate *pAc5-bioSUMO-BirA*, degenerated *Drosophila smt3* sequence (EMBL database accession number FN539078) [110] was fused to a biotin tag according to the strategy described [113]. The fusion was cloned into the *pAc5-FLAGmCherry-BirA* vector by substituting *FLAGmCherry*.

*Drosophila* S2R+ cells were obtained from DGRC [114]. Cells were cultured at 25°C in *Drosophila* Schneider's medium (Invitrogen) supplemented with 10% fetal bovine serum (Gibco) and 1% penicillin/streptomycin (Gibco). Transfections were performed using Effectene (Qiagen) in 6-well plates with 1 µg of *pAC5-Gal4* (Addgene #24344) [115], 1 µg of *pUASTattB-Flag-βftz-f1* and 1 µg of *pAc5-bioSUMO-BirA* or *pAc5-BirA*.

Transfected cells were collected after 3 days, washed with phosphate buffered saline (PBS) 1X and lysed in 200 µl of lysis buffer [8 M urea, 1% SDS, 50 mM N-ethylmaleimide in PBS and protease inhibitor mixture (Roche)]. Pulldowns were done according to [113] using 50 µl suspension of NeutrAvidin-agarose beads (ThermoScientific). For elution, samples were heated 5 minutes at 95°C in 4× Laemmli sample buffer with 100 mM DTT. The eluted sample was separated from the beads using a Vivaclear Mini 0.8 µm PES microcentrifuge filter unit. The recovered volume for both control and experimental samples was 30 µl.

For Western blots we used mouse monoclonal anti-Flag M2 antibody (1∶2000; Sigma), HRP-conjugated secondary antibody (1∶5000; Jackson ImmunoResearch) and HRP-linked anti-Biotin antibody (1∶200; Cell Signaling Technology).

### RNA probe preparation and *in situ* hybridization

ESTs from the DGRC cDNA collections were used as templates for the synthesis of the RNA probes (IP13851 for *Snmp1*, RE21078 for *pes*, RE02070 for *crq* and LD34889 for *ftz-f1*). RNA labeling was performed using the DIG RNA labeling Mix (Roche) according to the manufacture instructions, using 1 µg of linearized DNA.

RNA probes were hybridized to larval tissues at 55°C in 50% deionized formamide (Sigma), 5× saline sodium citrate, 50 µg/ml heparin sodium salt (Sigma), 0.1% Tween 20, and 100 µg/ml of phenol extracted sonicated salmon sperm DNA (Amersham Biosciences). Samples were incubated with anti-digoxigenin antibody (1∶2000; AP Fab fragments, Roche) and signal was detected using 4-Nitro blue tetrazolium chloride and 5-Bromo-4-chloro-3-indolyl-phosphate (Roche).

### qPCR analysis

RNA was extracted from isolated ring glands complexed with brain hemispheres placed in RNAlater (Ambion) and frozen in liquid nitrogen. At least two different pools of 50 to 100 specimens were collected per genotype. Total RNA extracts were obtained using the “mirVana miRNA isolation kit” (Ambion) according to the manufacturer's instructions and were quantified using Nanodrop (Thermo Scientific). cDNAs were prepared from 0.2 µg of RNA using the SuperScript III First-Strand Synthesis System for RT-PCR (Invitrogen) at a 10 µl volume per reaction, following manufacturer's instructions.

Oligonucleotides for *pes*, *crq*, *Snmp1* and *RpL32* were designed using NCBI primer blast (http://www.ncbi.nlm.nih.gov/tools/primer-blast). RpL32 was used as control. Oligo sequences were:

Pes(+)384Fwd, 5′-TCGCCGCTGCCTTTAGACTTCGATA-3′;

Pes(−)660Rev, 5′-CACGTCTAGCAGCAGAGTGCGCTAC-3′;

Crq(+)1747Fwd, 5′-GAGCCCGATGACGACTTCGACATAT-3′;

Crq(−)1967Rev, 5′-ACCCACTTTTTCGTCACAGTCAGCG-3′;

Snmp1(+)741Fwd, 5′-ATGGGTCAGGCCAATCACTCGGATT-3′;

Snmp1(−)935Fwd, 5′-CAGGCCATCCTCCTTTTTCAAGCCC-3′;

RpL32(+)365For, 5′-CCTTCCAGCTTCAAGATGACCATCC-3′;

RpL32(−)598Rev, 5′-ATCCGTAACCGATGTTGGGCATCAG-3′.

qPCR was done using FastStart Universal SYBR green Master (Roche). Reactions were performed in 10 µl, adding 2 µl of cDNA, 2× SYBR green and 0.2 µl of each primer (10 µM), in a CFX96-thermocycler (BioRad) with the following protocol: 95°C for 10 min, 40 cycles of 95°C for 10 seconds and 58.5°C for 1∶30 min, and a final extension of 95°C for 1 min. Per each pair of primers a melt curve from 65 to 95°C, with 0.5°C temperature increment every 5 seconds was done. Reactions were done in triplicates and checked by electrophoresis for validation of amplification specificity. RpL32 was used as control.

### Luciferase assay

*Drosophila* S2R+ cells were seeded in 24-well plates and transfected with 150 ng of *pSnmp1-FLuc* or *pSnmp1Mut-FLuc*, either alone or co-transfected with the same quantities of *pUASTattb-αftz-f1*, *pUASTattb-βftz-f1*, *pUASTattb-Smt3-αftz-f1* or *pUASTattb-Smt3-βftz-f1*. 150 ng of *pAc-Gal4* [116] and 50 ng of *pAc-Renilla* were added to all the wells. Transcriptional activity was measured 48 hours after transfection using the Dual-Luciferase Reporter Assay System (Promega), following the manufacturer's instructions. Luminescence was measured in a microplate luminometer (Veritas). Results are given as means+S.D. Differences between groups were calculated using Student's t test in Microsoft Excel.

### Immunocytochemistry

Adults were allowed to lay eggs during 8 hours and wandering larvae were collected 5 days AEL. Larvae and ovaries from adult flies were dissected in PBS, fixed in 4% paraformaldehyde for 20 minutes and washed three times in PBT (PBS, 0.3% triton X-100) for 20 minutes. Samples were blocked in PBT +1% BSA for one hour and incubated with the appropriate antibodies at 4°C overnight. Next day, tissues were washed with PBT three times, for 20 minutes each and incubated with secondary antibodies at room temperature for two hours. The following primary antibodies were used: guinea pig polyclonal anti-Cad99C (1∶3,000) [47]; mouse monoclonal DE-Cad (1∶25; DCAD2, Developmental Studies Hybridoma Bank); rabbit polyclonal anti-Smt3 (1∶500) [117]; goat polyclonal anti-Ftz-f1 (1∶25; Santa Cruz, Sc-27221); rabbit polyclonal anti-Crq (1∶100) [118]; rabbit polyclonal anti-Snmp1 (1∶1000) [119] and rabbit polyclonal anti-Dib (1∶200) [26]. Fluorescence Alexa 568 secondary antibody (Molecular Probes) was used at 1∶200 dilution. DAPI (Roche) was used at 1∶2000 dilution. Phalloidin-TRITC (Sigma) was used 1∶1000. Samples were mounted in Vectashield (Roche) mounting medium. Confocal images were taken with a Leica DM IRE2 confocal microscope.

### Oil Red O stainings

Ring glands were fixed in 4% paraformaldehyde for 20 minutes, washed twice in PBS and stained with Oil Red O (Sigma) solution at 0.06% in isopropanol for 30 minutes. Samples were washed twice in PBS before mounting in Vectashield. Images were taken with a Leica DM IRE2 confocal microscope.

Quantification of lipid droplets was done on single plane confocal micrographs of Oil Red O stainings using the Analyze Particle tool from ImageJ software. At least 10 independent micrographs were analyzed per genotype. Measurements were analyzed and plotted using Microsoft Excel.

### 20E feeding experiments

*phm-Gal4>UAS-ftz-f1i* and *phm-Gal4>UAS-Snmp1i* larvae were collected at 120 hours AEL and placed in groups of 10 individuals in new tubes. These were supplemented with 20E (Sigma) dissolved in ethanol and mixed with yeast at 1 mg/ml. Control larvae were fed with yeast supplemented with ethanol alone.

## Supporting Information

Figure S1 Conservation of SUMOylation consensus sites in Ftz-f1 related sequences from vertebrates to insects. ClustalW analysis of the Ftz-f1 homologues in the indicated species. SUMOylation consensus sites conserved only among insects are marked in pink, those conserved between insects and NR5A2 homologues are highlighted in orange and those conserved among the vertebrate NR5A2 and NR5A1 (SF-1) are indicated in blue. Orange and purple asterisks mark the sites SUMOylated in rat LRH-1/NR5A2 [14]. Blue and purple asterisks indicate the sites SUMOylated in mouse SF-1 [21]. The blue bar above the alignments indicates the DNA binding domain, while the red bars indicate the ligand binding domains. Below the alignments, asterisks indicate identical amino acids, colons indicate conserved substitutions and periods indicate semiconserved substitutions. Accession numbers of the sequences used for the analysis: *Aedes aegypti*, XP_001654601.1; *Anopheles gambiae*, XP_315680.4; *Apis mellifera*, XP_001122182.2; *Blattella germanica*, CAQ57670.1; *Bombyx mori*, BAK53999.1; *Drosophila melanogaster*, NP_524143.2; *Gallus gallus* NR5A2, NP_990409.1 and SF1, BAA76713.1; *Homo sapiens* NR5A2, NP_003813.1 and SF-1, NP_004950.2; *Manduca sexta*, AAL50351.1; *Monodelphis domestica* NR5A2, XP_001377433.2 and SF-1, XP_001371703.2; *Mus musculus* NR5A2, NP_109601 and SF-1, NP_620639.1; *Rattus norvegicus* NR5A2, NP_068510 and SF-1, NP_001178028.1; *Tribolium castaneum*, XP_970369.2; *Xenopus laevis* NR5A2, NP_001081185.1 and SF-1, NP_001091438.1.
(TIF)

Figure S2 Expression of Scavenger Receptors in PGs of WT or *smt3i* backgrounds. (A–H) Micrographs of *crq* (A–D) or *pes* (E–H) mRNA *in situ* hybridization in PGs from WT (A, B, E, F) or *phm-Gal4>UAS-smt3 RNAi* (*smt3i*) larvae (C, D, G, H) at the indicated hours AEL. *crq* mRNA is upregulated in WT clear-gut larvae (B, compare with A). However, no expression is visible in *smt3i* PGs (C, D). *pes* mRNA is also moderately upregulated in WT clear-gut larvae (B, compare with A) but, in contrast to *crq* and *Snmp1*, is still expressed in *smt3i* larvae (G, H). All the *in situ* reactions were stopped at the same time and pictures were taken at the same magnification.
(TIF)

Figure S3 Distribution of lipid droplets when *smt3* or *Snmp1* are silenced. (A–B) Single plane confocal micrographs taken under the same intensity settings showing the lipid droplets marked by Oil Red O staining (green). Nuclei are labelled with DAPI (purple). F-actin cytoskeleton is shown in blue. Follicle cells are indicated by yellow dotted lines. (A′–B′) Single green channels are shown in black and white. (A) In *T155-Gal4>UAS-smt3i* follicle cells show reduced lipid droplets. However, the oocyte (its nucleus in purple indicated by a yellow arrowhead) gets lipids through the ring canals (white arrowhead) from the nurse cells. Lipids are indicated by yellow arrowheads in A′. (B) In *T155-Gal4>UAS-Snmp1i*, the lipid intake by follicle cells is reduced. Droplets only occupy the basal side of the cells (b; indicated by a yellow arrowhead), while the apical side remains depleted (a).
(TIF)
