# Supplementary figures and images for: Scavenger Receptors Mediate the Role of SUMO and Ftz-f1 in Drosophila Steroidogenesis
Source: PLoS Genet. 2013 Apr 18;9(4):e1003473. doi: 10.1371/journal.pgen.1003473 (PMC3630131; doi:10.1371/journal.pgen.1003473)

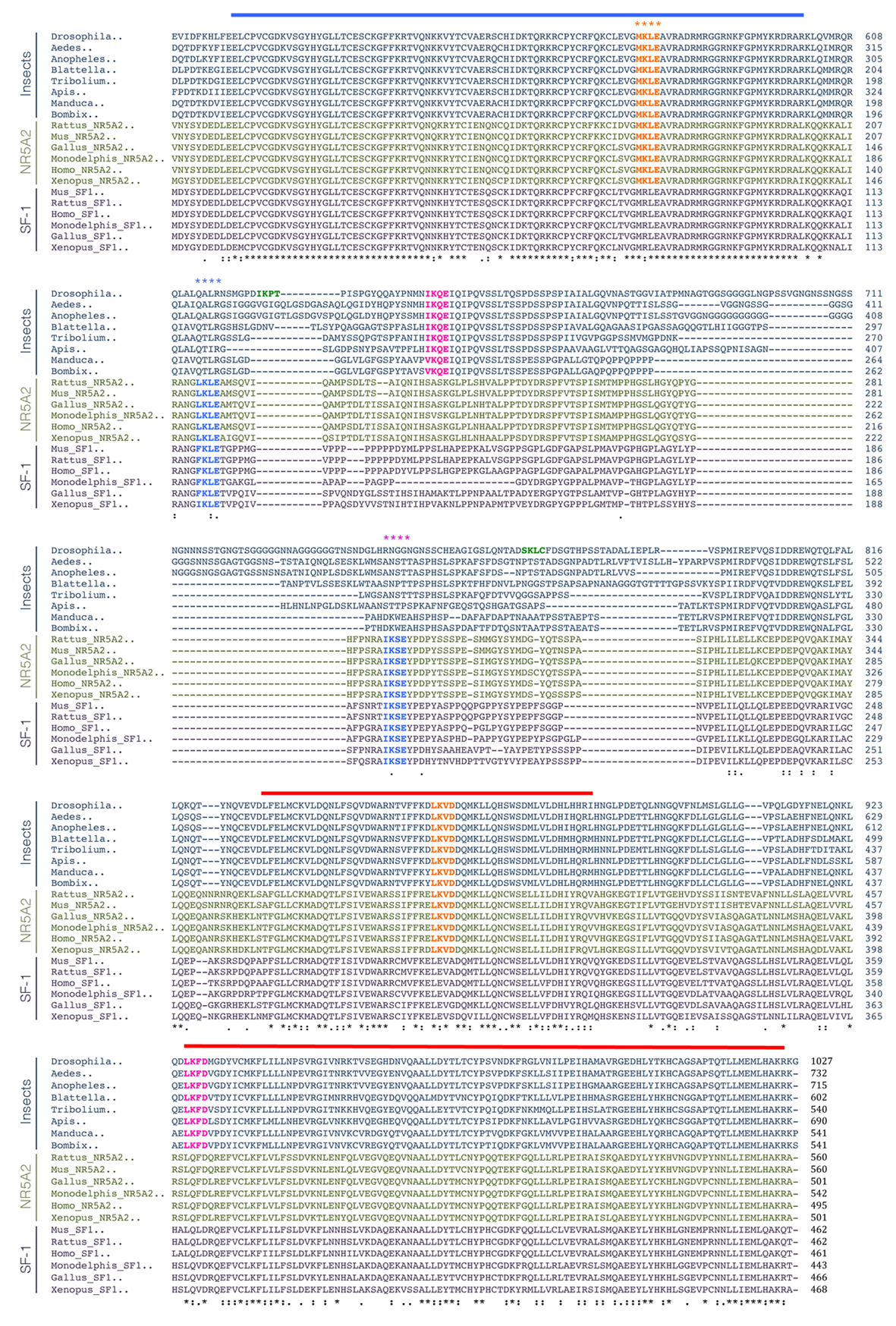

Supplement: Figure S1 — Conservation of SUMOylation consensus sites in Ftz-f1 related sequences from vertebrates to insects. ClustalW analysis of the Ftz-f1 homologues in the indicated species. SUMOylation consensus sites conserved only among insects are marked in pink, those conserved between insects and NR5A2 homologues are highlighted in orange and those conserved among the vertebrate NR5A2 and NR5A1 (SF-1) are indicated in blue. Orange and purple asterisks mark the sites SUMOylated in rat LRH-1/NR5A2 [14]. Blue and purple asterisks indicate the sites SUMOylated in mouse SF-1 [21]. The blue bar above the alignments indicates the DNA binding domain, while the red bars indicate the ligand binding domains. Below the alignments, asterisks indicate identical amino acids, colons indicate conserved substitutions and periods indicate semiconserved substitutions. Accession numbers of the sequences used for the analysis: Aedes aegypti, XP_001654601.1; Anopheles gambiae, XP_315680.4; Apis mellifera, XP_001122182.2; Blattella germanica, CAQ57670.1; Bombyx mori, BAK53999.1; Drosophila melanogaster, NP_524143.2; Gallus gallus NR5A2, NP_990409.1 and SF1, BAA76713.1; Homo sapiens NR5A2, NP_003813.1 and SF-1, NP_004950.2; Manduca sexta, AAL50351.1; Monodelphis domestica NR5A2, XP_001377433.2 and SF-1, XP_001371703.2; Mus musculus NR5A2, NP_109601 and SF-1, NP_620639.1; Rattus norvegicus NR5A2, NP_068510 and SF-1, NP_001178028.1; Tribolium castaneum, XP_970369.2; Xenopus laevis NR5A2, NP_001081185.1 and SF-1, NP_001091438.1. (TIF) [file pgen.1003473.s001.tif]

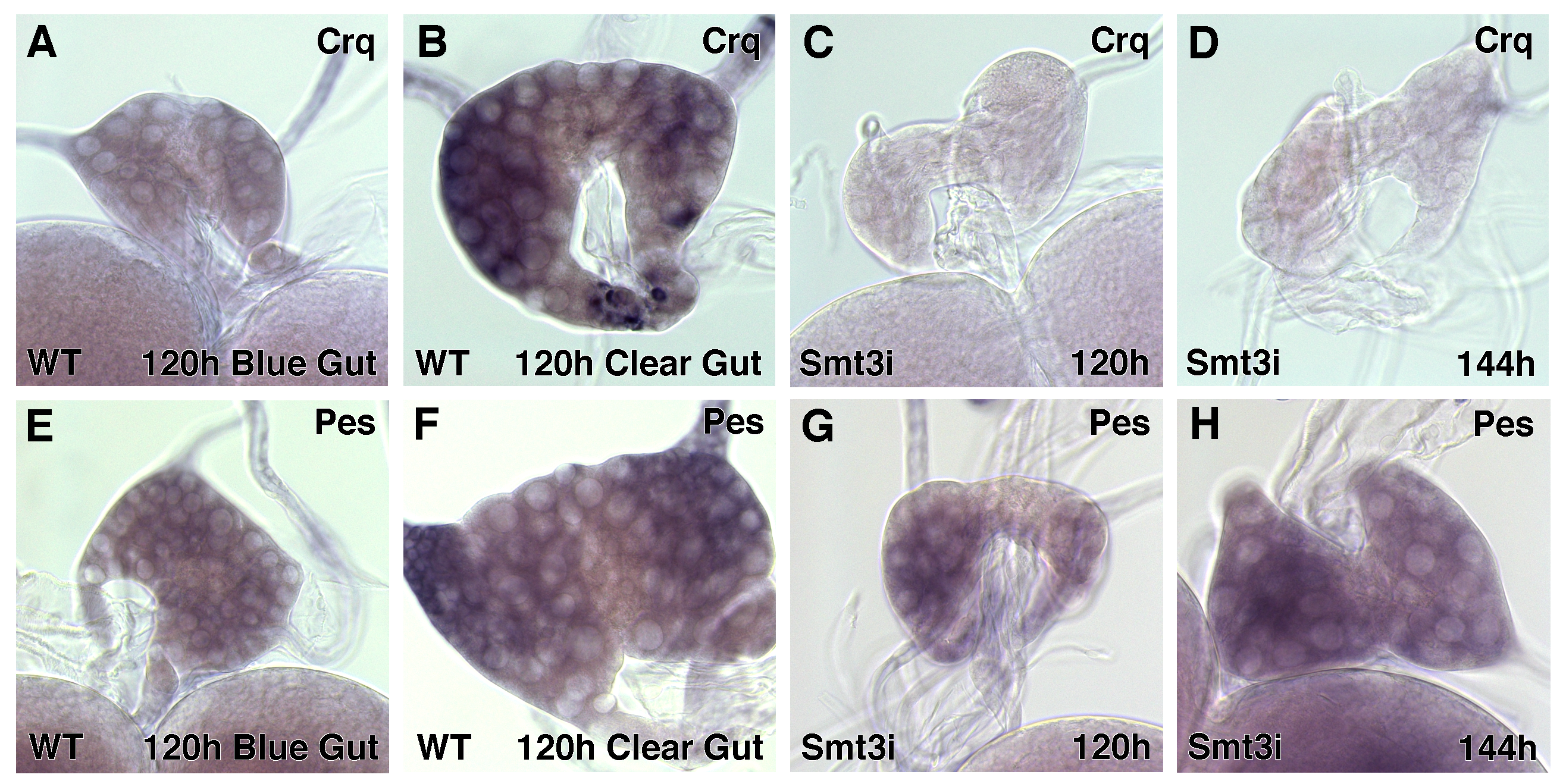

Supplement: Figure S2 — Expression of Scavenger Receptors in PGs of WT or smt3i backgrounds. (A–H) Micrographs of crq (A–D) or pes (E–H) mRNA in situ hybridization in PGs from WT (A, B, E, F) or phm-Gal4>UAS-smt3 RNAi (smt3i) larvae (C, D, G, H) at the indicated hours AEL. crq mRNA is upregulated in WT clear-gut larvae (B, compare with A). However, no expression is visible in smt3i PGs (C, D). pes mRNA is also moderately upregulated in WT clear-gut larvae (B, compare with A) but, in contrast to crq and Snmp1, is still expressed in smt3i larvae (G, H). All the in situ reactions were stopped at the same time and pictures were taken at the same magnification. (TIF) [file pgen.1003473.s002.tif]

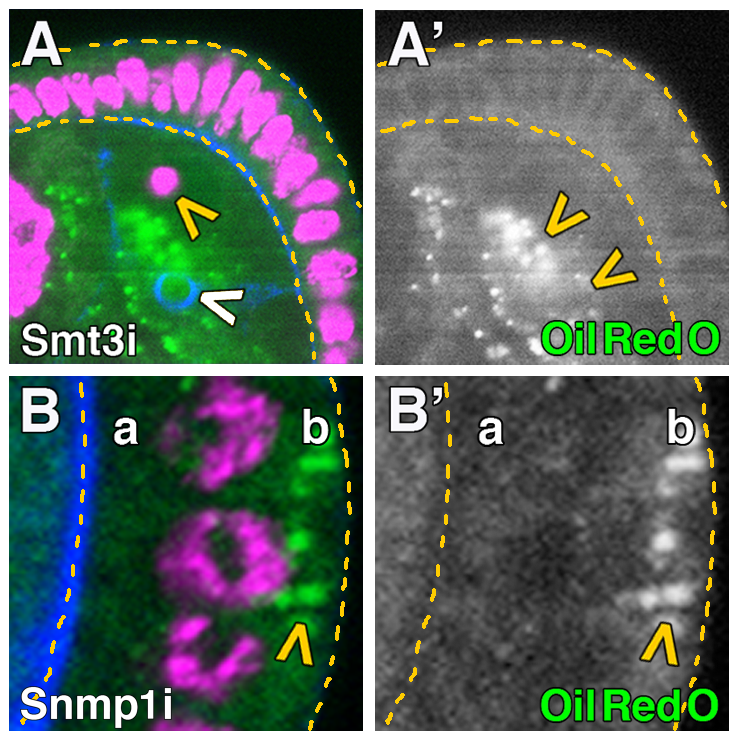

Supplement: Figure S3 — Distribution of lipid droplets when smt3 or Snmp1 are silenced. (A–B) Single plane confocal micrographs taken under the same intensity settings showing the lipid droplets marked by Oil Red O staining (green). Nuclei are labelled with DAPI (purple). F-actin cytoskeleton is shown in blue. Follicle cells are indicated by yellow dotted lines. (A′–B′) Single green channels are shown in black and white. (A) In T155-Gal4>UAS-smt3i follicle cells show reduced lipid droplets. However, the oocyte (its nucleus in purple indicated by a yellow arrowhead) gets lipids through the ring canals (white arrowhead) from the nurse cells. Lipids are indicated by yellow arrowheads in A′. (B) In T155-Gal4>UAS-Snmp1i, the lipid intake by follicle cells is reduced. Droplets only occupy the basal side of the cells (b; indicated by a yellow arrowhead), while the apical side remains depleted (a). (TIF) [file pgen.1003473.s003.tif]
